# Small-area geographical variation in the prevalence of diabetes amongst Australian youth aged <20 years in 2021

**DOI:** 10.1016/j.anzjph.2025.100234

**Published:** 2025-06

**Authors:** Ewan Cameron, Song Zhang, Aveni Haynes, Peter W. Gething

**Affiliations:** 1 Geospatial Health and Development Team, Telethon Kids Institute, Perth, 6009, Australia; 2 School of Public Health, Curtin University, Perth, 6102, Australia; 3 Stan Perron Foundation Fellow, Australia; 4 Children’s Diabetes Centre, Telethon Kids Institute, the University of Western Australia, Perth, 6009, Australia

**Keywords:** diabetes mellitus, type 1—juvenile-onset diabetes, epidemiologic factors, spatial regression

## Abstract

**Objectives:**

To characterise small-area geographical variation in the prevalence of diabetes in Australian youth.

**Methods:**

A combined statistical reconstruction and small-area estimation algorithm was applied to privacy-modulated data from the 2021 Australian Census. The census instrument and reconstruction accuracy was examined by comparisons against a hospital-based register and community register. Diabetes prevalence maps were created from the small-area estimates.

**Results:**

The median and interquartile range of estimated diabetes prevalence by small-area unit under our geospatial smoothing model were 1.76 [1.49–1.97] cases per 1000 population for those aged 0–14 years and 5.2 [4.4–5.9] cases per 1000 population for those aged 15–19 years old. Concentrations of elevated prevalence were identified in the vicinities of regional towns across South-East Queensland, regional New South Wales and regional Victoria. Across each of Australia’s five largest cities a gradient of decreasing youth diabetes prevalence from the outer suburbs to the urban centre was identified.

**Conclusion:**

Diabetes burden is systematically higher among rural and peri-urban resident youth in Australia compared with their urban counterparts.

**Implications for Public Health:**

Hotspots of prevalence in regional areas deserve attention from public health authorities.

## Introduction

Spatial analyses of variation in disease burden can offer valuable insights for epidemiologists and public health policymakers.^1^ Geostatistical demarcations of high and low burden areas can serve to test and/or generate hypotheses regarding environmental risk factors and may also guide decision-making towards the improved alignment of service provision with community needs and the reduction of health inequalities.^2^

International variations in the incidence rate of type 1 diabetes were early signifiers of the role of genetic susceptibility and environmental factors such as vitamin D sufficiency in this autoimmune disease.^3^^,^^4^ Sub-national variations in type 1 diabetes burden have also been used to explore the ‘hygiene hypothesis’: namely, the proposition that stimulation of the immune system by exposure to common infections in early life has a protective effect.^5^ Spatial regression analyses in Northern Ireland and Spain have reported higher incidences in sparsely populated, rural areas and lower incidences in more densely populated, urban areas,^6^^,^^7^ but the opposite has also been observed in Western Australia.^8^ Small-area analyses of type 2 diabetes cases in Germany and the United States have revealed a strong relationship with obesity and area deprivation^9^ and no relationship with outdoor air pollution.^10^

A lack of nationally representative data with fine-scale geographical specification has been a limiting factor for small-area spatial analyses of the diabetes burden in Australia. The aforementioned study in Western Australia was based on the Western Australian Children's Diabetes Database (WACDD). The WACDD was established in 1987 at the Princess Margaret Hospital (now Perth Children’s Hospital) and continues to collect information on all consenting patients attending the hospital-based diabetes clinic, which is the only such facility in the state. The completeness of the WACDD has been estimated at over 99% for type 1 and over 70% for type 2 by capture-recapture methods.^11^^,^^12^ The overwhelming concentration of Western Australia’s population in the southwest of the state limits the power of this dataset for identifying the demographic and environmental factors influencing spatial variation.

At the national level, there is the voluntary register of the National Diabetes Services Scheme (NDSS), which is estimated to hold information on 80–90% of Australians with insulin-treated diabetes.^13^ Censored counts of registrants in ten-year age groups are made publicly available across variety of spatial unit systems with the smallest being the postcode level; censoring is applied where there are fewer than 20 registrants in an area unit and thus most heavily impacts the postcode level dataset. A recent spatial analysis of all-age type 2 diabetes using the NDSS encountered missing data due to censoring across 26% of Australian postcodes.^14^

In 2021, a new question on chronic health conditions was added to the Australian Census with diabetes included among a list of disease-specific response items. The Australian Census is a whole-of-population survey and the overall response rate for the long-term health conditions question was 91.9%.^15^ Data summaries and insights from the Australian Census are made available to researchers by the Australian Bureau of Statistics (ABS) through the TableBuilder platform. The geographical subdivisions of this dataset include those of the Australian Statistical Geography Standard (ASGS), which form a nested hierarchical system spanning a wide range of spatial scales. Although noise injection and low-count suppression are applied with TableBuilder to protect respondent privacy,^16^ the nested structure of the ASGS hierarchy may be leveraged to achieve effective statistical reconstruction at the finest scales with modern Bayesian methods.^17^ The analysis of the 2021 Australian Census thus offers an unprecedented opportunity to explore small-area spatial trends in youth diabetes prevalence across the whole of Australia.

## Methods

### Diabetes datasets

Age-structured counts of persons by diabetes status—across the three categories of the Australian Census 2021 diabetes item (HDIAP): ‘Has diabetes (excluding gestational diabetes)’, ‘Does not have diabetes (excluding gestational diabetes)’ and ‘Not Stated’—were extracted from the ABS TableBuilder platform. It is important to note that this question does not distinguish between type 1 and type 2 diabetes. Queries were made at each of the ASGS subdivisions from SA1 (approximately 200–800 people) to SA4 (over 100,000 people), as well as the state/territory and National totals. Summaries of HDIAP class counts by socioeconomic status (‘Index of Relative Socioeconomic Advantage and Disadvantage’ decile; IRSAD) and remoteness (Remoteness Area: five categories) were also requested. Demographic filtering was applied in TableBuilder to select residents in each of the three age groups: 0–9, 10–14 and 15–19 age. These Census extracts represent the primary dataset against which we apply our statistical reconstruction and small-area estimation procedures.

For comparison purposes, counts by SA3 unit of children diagnosed with type 1 or type 2 diabetes aged 0–9 and 10–14 years old as of the 2021 Census month of August were extracted from the WACDD. The authors’ access to this dataset for research purposes was approved by the Child and Adolescent Health Service Human Research Ethics Committee, Western Australia (RGS0000002386). Also for comparison purposes, counts of insulin-dependent diabetes registrants were extracted from the NDSS Diabetes Map tool in the two available age groups of 0–9 and 10–19 year of age at the Local Government Area (LGA) spatial scale, along with the state/territory totals. In addition to the censoring of counts below 20, a rounding of all counts to the nearest ten is applied in the NDSS map to further safeguard registrant privacy.^18^

### Statistical model

Reconstruction of the true Census counts by HDIAP category proceeded under a Bayesian model-based algorithm; the development of this algorithm and its performance against simulated data are described in depth in an earlier manuscript.^17^ In brief, a Markov Chain Monte Carlo procedure was used to explore the range of true cell counts consistent with the available TableBuilder outputs in each ASGS unit system (SA1, SA2, SA3, SA4, and STE), as well as the socioeconomic and remoteness categories, under a simple model for the TableBuilder perturbation process. A blocked Gibbs sampling scheme was applied within this algorithm to achieve feedback against a geospatial smoothing model for the proportion positive. A full mathematical description of the smoothing model and details of its computational implementation are given in the Supplementary Information and we summarise only its key features below.

The expected proportion of children with diabetes in each SA1 unit in the youngest and oldest age groups 0–9 and 15–19 years old is modelled separately under a logit transform as the sum of a constant intercept term, a socioeconomic effect, a remoteness area effect, and a spatial random field; the latter encodes the geographical assumption that nearby areas are likely to have similar prevalence. The expected proportion of children and early adolescents with diabetes aged 10–14 years old in each SA1 area is modelled as a mixture of the models for the youngest and oldest age groups with the mixing proportion treated as a parameter to be learned during model fitting. Areal aggregation matrices built from ABS correspondence tables connect the reconstructed SA1 totals and expectations to their WACDD and NDSS counterparts at the SA3 and LGA levels for post-fit comparisons.

Data processing and statistical analysis were performed using R (version 4.4.0) with Template Model Builder and the Integrated Nested Laplace Approximation (the ‘TMB’ and ‘INLA’ packages). The R code and TMB file used for this analysis are made publicly available online at https://github.com/drewancameron/diabetesmodel .

## Results

### Fidelity of the ABS Census Data with the WACDD and NDSS

For Western Australia (queried at the State level) TableBuilder reports totals of 302, 498 and 712 respondents answering in the affirmatively to the diabetes prompt under the long-term health conditions question in the age groups of 0–9, 10–14 and 15–19 years old, respectively. In comparison the WACDD register lists totals of 285 (284 type 1; 1 type 2) and 384 (360 type 1; 24 type 2) children aged 0–9 and 10–14 years old as of the date of the Census 2021. The NDSS queried (in October 2023) at the state/territory level reports 310 and 1410 registrants aged 0–9 and 10–19 years old, respectively. The closest agreement between the three datasets is thus for the youngest age group, and we note that these cases are expected to be almost exclusively type 1 diabetes. For older children and young adults the differences are more substantial: the WACDD count for 10–14-year-old children is only 77% of that in the Census and the Census 10–19-year-old youth total (1210) is only 86% of the corresponding NDSS total. Double registration of individuals who change states of residence was noted as a potential problem for the NDSS dataset in a report prepared by the AIHW,^19^ along with the missing data of those self-managing type 2 diabetes through diet and exercise; however, this report was prepared in 2009 and data quality may well have improved since then.

**Figure 1:**
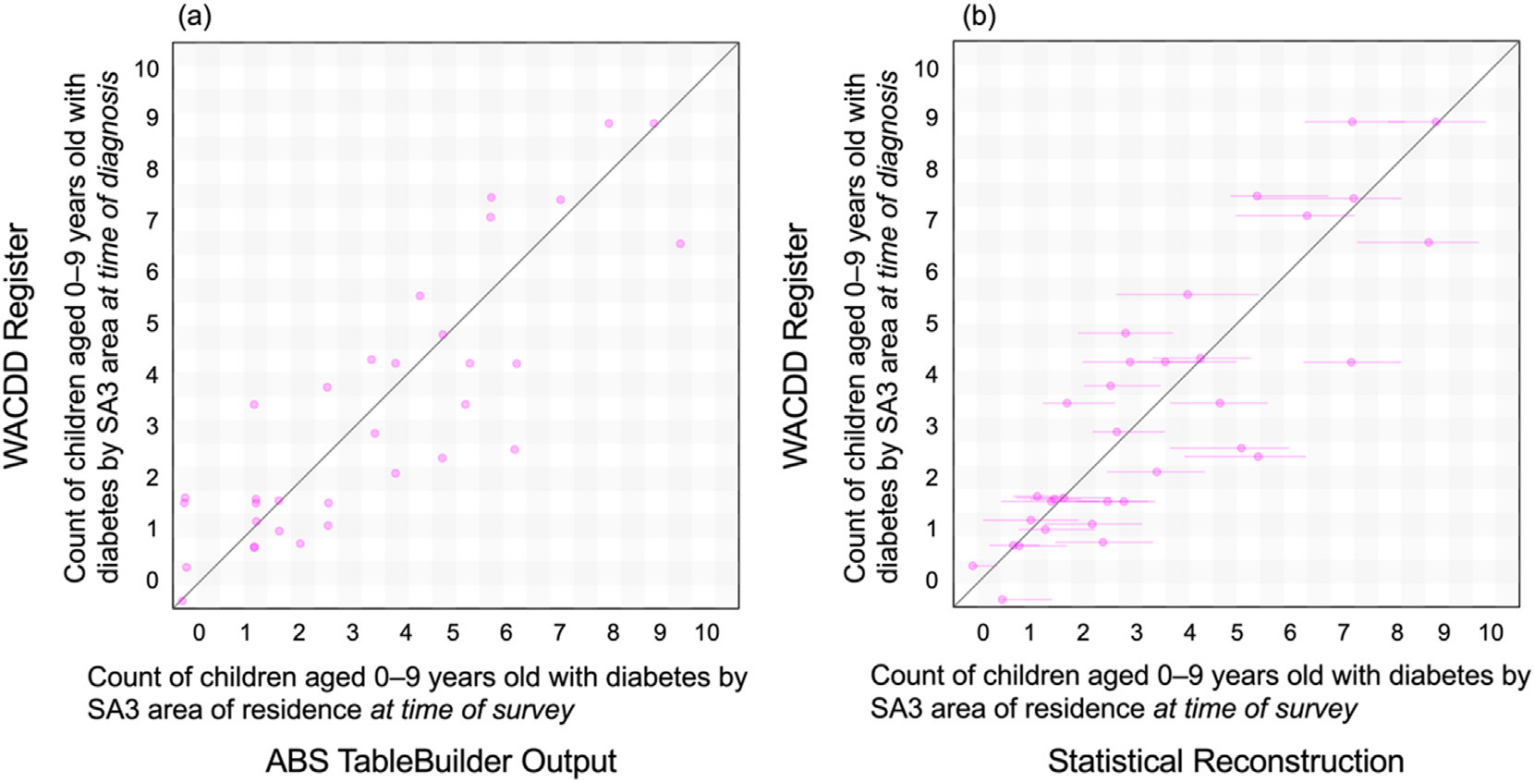
Counts of children with diabetes aged 0–9 years old by SA3 unit in the ABS Census 2021 compared against those in the WACDD. The address information against which the SA3 unit is assigned is that of residence at the time of survey for the ABS Census and in the WACDD it is residence at time of diagnosis. In Panel (a) the ABS Census datum used in the comparison is the raw (i.e., noise-injected and low count suppressed) TableBuilder output while in Panel (b) we show the posterior median count and 95% credible interval produced under our statistical reconstruction algorithm.

In Panel A of Figure 1, we present graphical comparisons of the counts of 0–9-year-old children with diabetes by SA3 unit according to ABS TableBuilder and the WACDD register. Note that the ABS areal unit membership is based on the current place of usual address at the time of the 2021 Census, while that in the WACDD is based on patient address at time of diagnosis. In Panel B, we repeat this comparison using our reconstructed counts based on the ABS data extracted across all ASGS spatial scales. The impact of the reconstruction procedure is seen where non-zero values are imputed for some SA3 units that had zero TableBuilder outputs. Likewise, the counts assigned to some of the SA3 units that had non-zero TableBuilder outputs are curbed through the effect of Bayesian shrinkage. The reconstructed estimates are marked as points at their posterior median estimates with error bars indicating their corresponding 95% credible intervals. The correlation coefficient between the WACDD counts and the raw TableBuilder outputs is 0.88, and that between the WACDD count and the reconstructed counts is identical.

**Figure 2:**
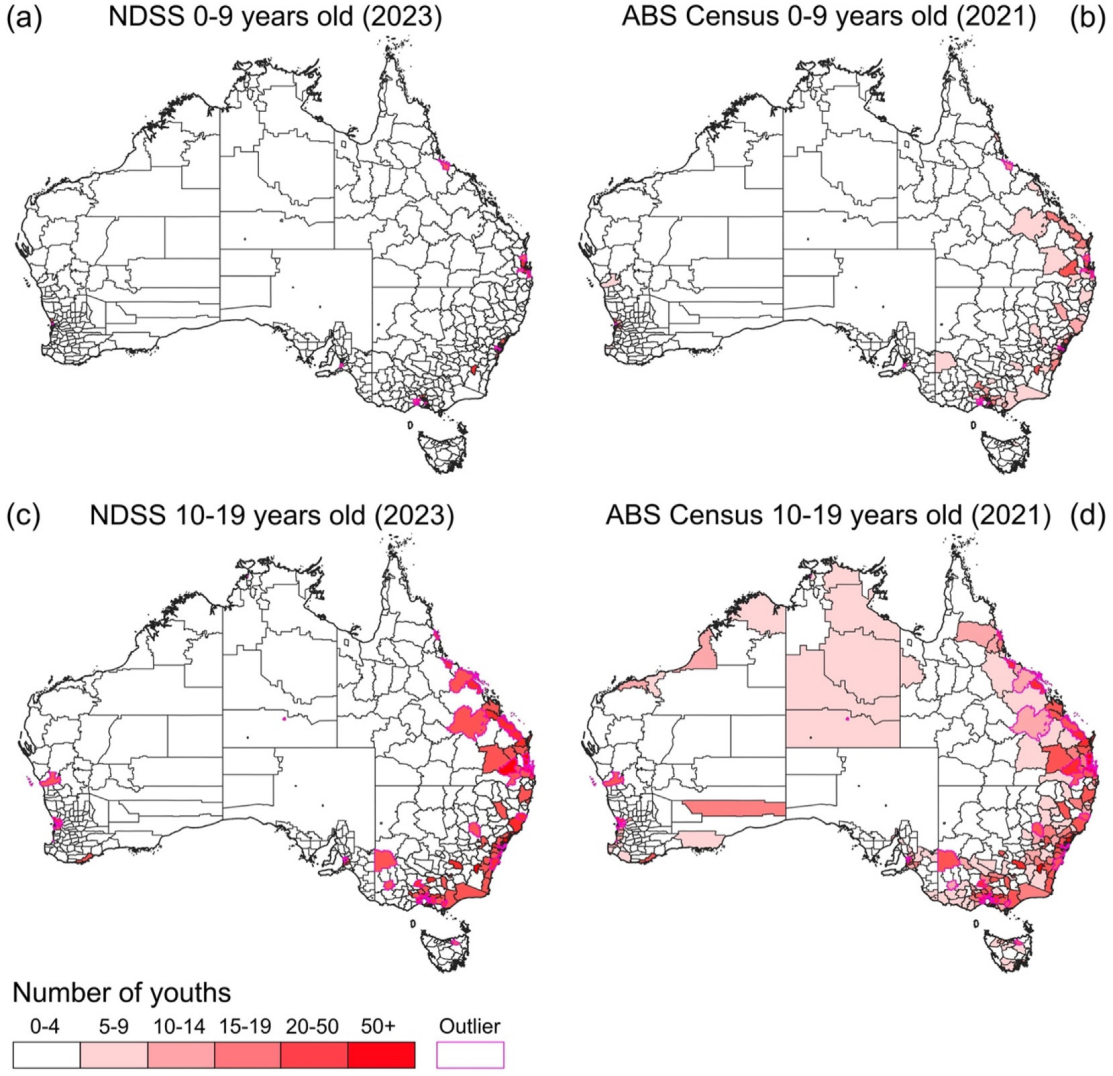
Counts of youths with diabetes by LGA unit in the NDSS (2023) compared against those from reconstruction of the ABS Census 2021. The comparison for children aged 0–9 years old is made between Panels (a) and (b), while that for children and adolescents aged 10–19 years old is made between Panels (c) and (d). The ABS Census datum used in these comparisons is the posterior median count from our statistical reconstruction procedure. A number of LGAs that were identified as outliers in testing of the posterior 95% credible interval of our statistical reconstructions against the reported NDSS values are highlighted with magenta borders. Suppressed NDSS outputs (where there are less than 20 registered children in that LGA) are plotted as zero counts here.

In Figure 2 we present maps comparing the NDSS reports by LGA against those from our reconstruction of the ABS Census data for youths aged 0–9 and 10–19 years old, respectively. The impact of drop-out due to censoring of counts less than 20 from the NDSS map is evident, with the ABS Census identifying non-zero case counts in many LGAs in both age groups. For the younger age group most of these LGAs where suppression is inferred to have removed significant case counts lie on the east coast, while for the older age group there are also large portions of the sparsely populated Northern Territory where count suppression has been impactful. We have highlighted in Figure 2 those LGAs where the posterior 95% credible interval of case counts from the ABS Census reconstruction does not include the NDSS map value; totals of 12 (2.1%) and 63 (11%) LGAs for children aged 0–9 years old and children and adolescents aged 10–19 years old, respectively.

Comparing the ABS Census totals against those from the NDSS map at the state/territory level for the 0–9-year-old age group we have 766 (ABS) vs 830 (NDSS) for New South Wales, 689 vs 750 for Victoria, 560 vs 570 for Queensland, 302 vs 310 for Western Australia, 193 vs 190 for South Australia, 71 vs 80 for Tasmania, 20 vs NA (0) for the Northern Territory and 44 vs 50 for the Australian Capital Territory. The equivalent comparison for the 10–19-year-old age group is 3494 (ABS) vs 3770 (NDSS) for New South Wales, 2746 vs 3080 for Victoria, 2505 vs 2970 for Queensland, 1210 vs 1410 for Western Australia, 887 vs 980 for South Australia, 272 vs 310 for Tasmania, 144 vs 160 for the Northern Territory and 211 vs 220 for the Australian Capital Territory. For the younger age group the overall total from the ABS (excluding the Northern Territory) is 94.4% of that from the NDSS, and for the older age group the corresponding proportion is 88.9%.

### Estimated spatial variation in childhood diabetes prevalence

**Figure 3:**
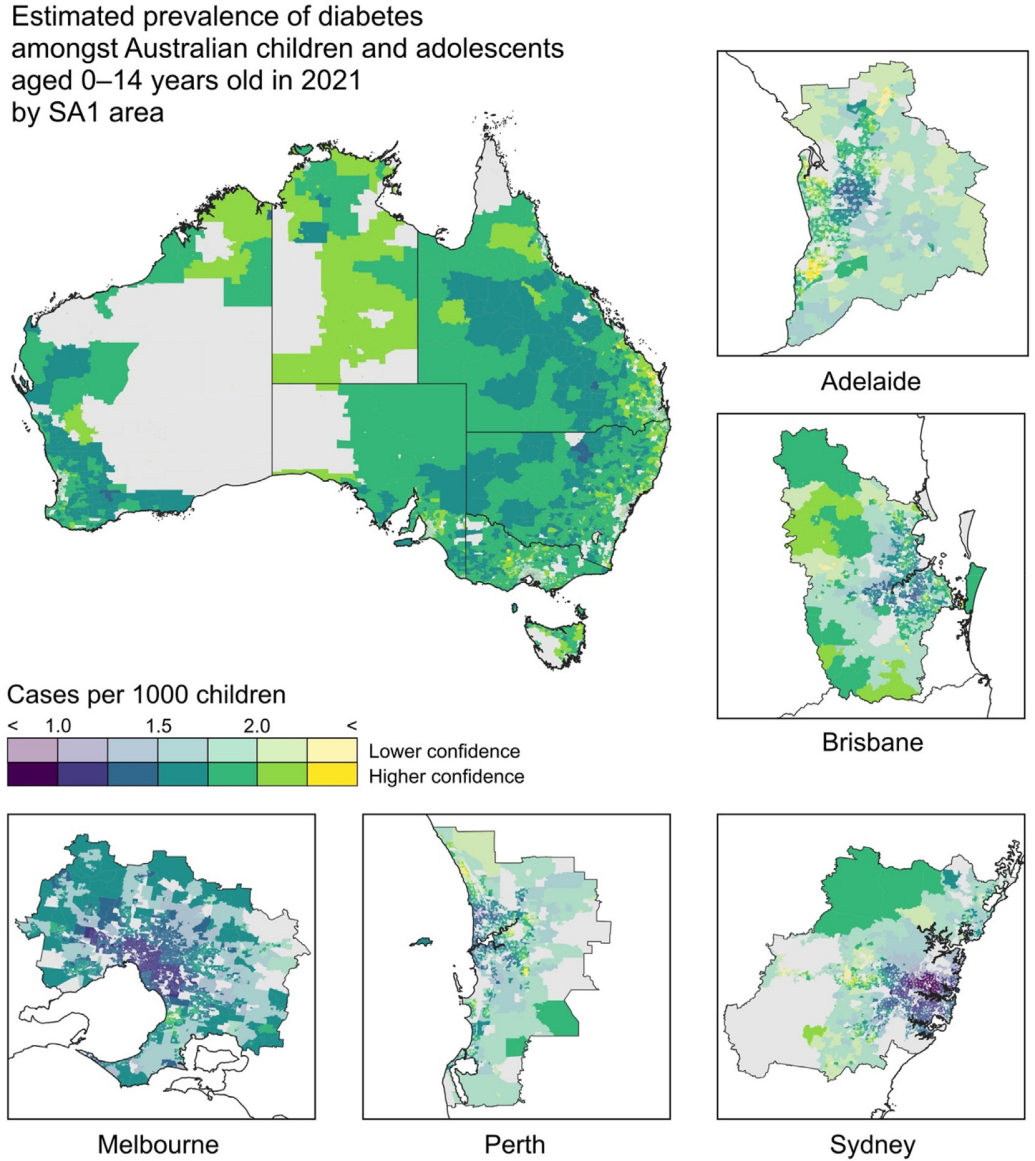
Posterior mean prevalence of diabetes among Australian children and early adolescents aged 0–14 years old by SA1 unit under our geospatial regression model. In addition to the national scale map, we highlight in panels the pattern surrounding each of Australia’s five largest cities.

**Figure 4:**
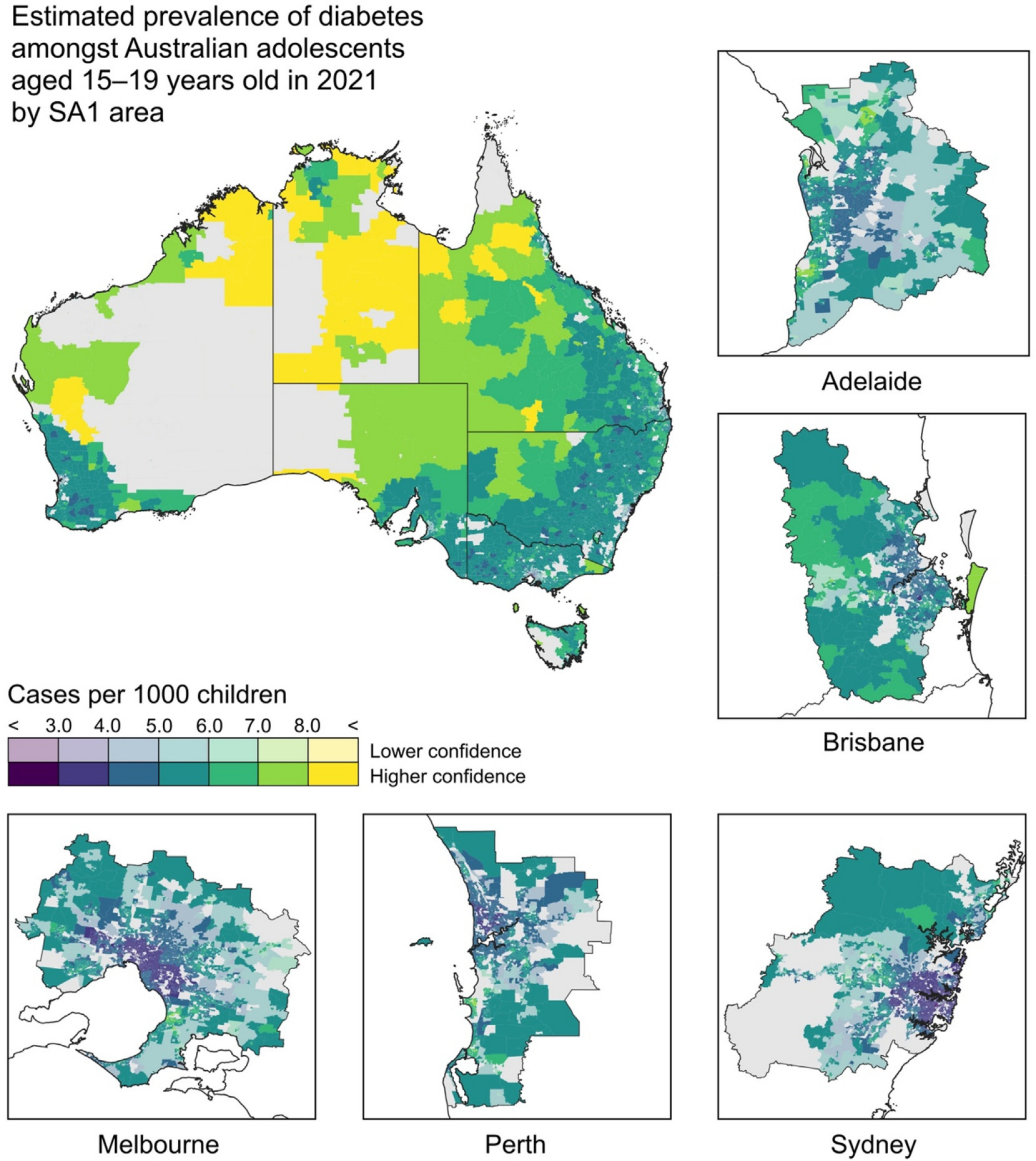
Posterior mean prevalence of diabetes among Australian adolescents aged 15–19 years old by SA1 unit under our geospatial model. In addition to the national scale map, we highlight in panels the pattern surrounding each of Australia’s five largest cities.

Under our small-area Bayesian geospatial smoothing model fit to the Australian Census 2021 dataset, we recover posterior median prevalences of 1.76 and 5.24 cases per 1000 population across SA1 units for Australian children and early adolescents aged 0–14 years old for and adolescents aged 15–19 years old, respectively. The corresponding inter-quartile ranges across SA1 units are 1.49–1.97 and 4.4–5.9 cases per 1000 population. The spatial pattern of diabetes prevalence is illustrated for the 0–14 year old age group in Figure 3 and for the 15–19 year-old-age group in Figure 4. To convey the level of uncertainty in these estimates we assign SA1 units to categories of lower and higher confidence. Higher confidence is defined here as a prevalence estimate for which the posterior standard deviation is less than 25% of the median prevalence.

The highest prevalence SA1 units for Australian children and early adolescents aged 0–14 years old are located in regional and peri-urban areas of the eastern States. ‘Hotspots’ appear across South-East Queensland and across regional New South Wales and regional. Elevated risk is also seen in the outer suburbs and satellite towns of the major cities. Conversely, the urban centres of Australia’s largest cities are identified as having substantially lower diabetes prevalences than their peri-urban surrounds. These spatial trends are closely reproduced, albeit at higher prevalences, in the 15–19 years old age group, but with one notable change: new areas of very high prevalence areas appear across the remote areas of the centre and north of Australia.

It is important to note is that the trends of increasing diabetes prevalence with decreasing socioeconomic status for 0–14 year olds, and additionally with increasing remoteness for 15–19 year olds, evident in Figure 3, Figure 4 are consistent with the summary data extracted along these dimensions from ABS TableBuilder and with the posterior estimates of the socioeconomic status and remoteness index effects in our model (reported in the Supplementary Information).

## Discussion

The inclusion of a long-term health conditions question for the first time within the Australian Census 2021 offers many new opportunities for the study of disease burden. In this analysis we have used statistical reconstruction and model-based inference procedures to characterise, for the first time, the small-area geographical variation in youth diabetes prevalence across Australia. We have thereby revealed a number of regional ‘hotspots’ of elevated prevalence around the country and have demonstrated a substantial gradient of decreasing prevalence from the peri-urban surrounds to the urban centres of Australia’s five largest cities.

### A note on the statistical model and dataset

Before reflecting further upon these results, it is important to note the expected impacts of the statistical model chosen for this analysis on the patterns in the disease maps so constructed. The use of spatial random fields within a Bayesian framework for smoothing and interpolating between noisy observations indexed to geographical locations or areal units is known as ‘model-based geostatistics’.^20^ The role of the random field component is to encode the geographical expectation that nearby locations will have similar demographic and environmental characteristics and therefore have similar levels of disease risk. The tendency of such models is to ‘shrink’ each area’s prevalence estimate towards a common mean, except where the data from neighbouring areal units substantively and collectively trend above or below the mean. However, this effect operates in practice only on residual variation not explained by our socioeconomic status and remoteness area effects, which themselves operate to ‘shrink’ estimates towards similarity along these dimensions of the dataset. The patterns that result in our maps are thus both a product of the data and the choice of model.

By restricting our model to the interaction between socio-economic status and remoteness effects without adding other covariates such as climatic variables we have created a parsimonious model that focuses estimation power on the intersection between these two fundamental stratifications of the Australian population. However, the posterior median maps we present clearly contain the imprint of this model structure and the true risk surface may in fact be more spatially smooth than this. At the same time, the spatial smoothing of the residual variability is conservative in terms of identification of ‘hotspots’ that do not align with socioeconomic or remoteness area trends. Hence, while we are confident in the existence of the ‘hotspots’ we have identified we caution that others may exist that are not discoverable with our choice of model and the current dataset.

Since the Australian Census dataset does not distinguish between type 1 and type 2 diabetes we refer to our model-based outputs simply as maps of childhood and adolescent diabetes. For children aged 0–9 years old we expect that the cases counted will be almost entirely type 1 diabetes, while for the older children and adolescents there will be a modest contribution from type 2 diabetes cases. While data on type 2 diabetes prevalence amongst Australian adolescents is limited, a recent study from the United States ^21^ offers insights into the probable fraction of cases here, with those authors reporting type 2 to type 1 ratios of 1:13 and 1:4.5 for the 10–14 and 15–19 years old age groups, respectively. Our decision to combine 0–9 and 10–14 year olds into one group and map these separately to the 15–19 years old age group was motivated by this expectation that the type 2 cases will likely only start to influence the fitted spatial pattern for this oldest age group. In our model, we included a free parameter to allowed the fitted prevalence for the 10–14 year old age group to be represented as a mixture between that of the 0–9 and 15–19 year old age groups, and the fitted parameter value of 98% (93-99%) strongly favoured the younger age group.

### Explaining the differences between datasets

While close agreements were observed between the case numbers in each dataset for children aged 0–9 years old there were notable discrepancies for the older age groups. The difference between the Census count for children aged 10–14 years old in Western Australia (498) and the WACDD count (384) is most likely due to missingess of community-managed type 2 cases in the WACDD. Completeness of type 1 diabetes case capture in the WACDD has been statistically estimated at 99.9% in a 2018 study^11^ and this estimate is thought to remain accurate since Perth Children’s Hospital is the only youth diabetes clinic in the State and all new onset cases are referred to the clinic. The completeness of type 2 diabetes case capture in the WACDD was previously estimated at 73% based on comparison with the NDSS over the period 1999–2016^12^ but it is possible this may have declined if screening and community-management has increased, particularly in remote locations. A study of 2016–2017 register data from primary healthcare services in Indigenous communities revealed very high prevalence rates of type 2 diabetes favouring the hypothesis of significant under-ascertainment in hospital-based registers.^22^ New screening and diagnosis guidelines designed to improve type 2 diabetes case detection have also been recently proposed by the Australasian Paediatric Endocrine Group.^23^

The differences in case counts for 10–19 years olds at the state/territory level in the Census compared with the NDSS (e.g. 1210 vs 1410 for Western Australia) are presumed to be the result of non-response in the census. The average non-response rate for the long-term health conditions question is 8.1%.^15^ However, this varies with population characteristics with higher non-response rates amongst low socioeconomic status and indigenous cohorts; both of which are also at elevated risk for type 2 diabetes. Exposure to non-response bias is thus a limitation of the present study with its possible impact being the non-detection of some additional high risk clusters.

### Explaining the spatial variation of childhood diabetes in Australia

The evidence presented here for a gradient of decreasing childhood diabetes towards the centres of our five largest cities is consistent with the results from multiple overseas studies; notably those small-area analyses from Northern Ireland^6^ and Spain^7^ mentioned in the Introduction. These trends are produced by a combination of the socio-economic effect (increasing prevalence with decline low socio-economic status) and the spatial random field in our model. At face value these trends could be explained by one aspect of the so-called ‘hygiene hypothesis’ whereby those living in densely populated urban centres would likely be exposed to an increased number of childhood infections resulting in a reduced risk of immune-mediated conditions in later life.^5^ However, the socio-economic effect also holds outside of these urban areas, with risk notably being higher in the regional towns of the eastern States than in their surrounding countryside.

As summarised in a recent literature review,^24^ the results of overseas studies examining the relationship between socioeconomic status and type 1 childhood diabetes risk show a variety of outcomes but overall offer little evidence for a consistent socioeconomic effect. The socioeconomic effect recovered in this study reproduces the empirical trend with socio-economic status seen in the ABS Census data for each age group (including that of 0–9 year olds which is expected to be almost entirely determined by type 1 diabetes) and indicates that it cannot be explained by confounding between remoteness and socio-economic status.

The regional areas where ‘hotspots’ of elevated prevalence were identified in our analysis include towns and cities with documented socio-economic factors, such as food insecurity,^25^ representative of risk under the ‘overload hypothesis’ for type 1 diabetes. Namely, that a high growth rate and/or stress in early life can overload the pancreatic beta cells and accelerate the development of auto-immune conditions.^26^ Again, however, there are significant spatial variations in population demographics (in particular, with regard to ancestry and Indigeneity that also covary spatially which must temper our interpretation of the maps presented here. Also worth noting is that there may be socio-economic and/or remoteness trends in diagnosis rates for type 2 affecting the older age group that contribute to shaping the observed trends.

### Possibilities for further insights with additional datasets

Given the relative rarity of childhood diabetes, complementary data will be required to power deeper investigations of the explanatory factors behind the spatial patterns observed here. Ideally, de-identified data with geographical, socioeconomic and demographic variables attached at the individual level rather than in aggregate as small-area counts to side-step the ‘ecological fallacy’,^27^ and with precise times of diagnosis to assist with causal attribution.^28^ The WACDD meets both of these criteria and is continuing to gather data, yet its spatial coverage is restricted to Western Australia. Researchers with access to individual-level data gathered by the NDSS or the multi-centre Australasian Diabetes Data Network (ADDN^29^) may also have the opportunity to contribute to the understanding of spatial variation, although no geographical analyses of non-public data from datasets have yet been published. Assuming that the long-term health conditions question is retained for the next Australian Census in 2026 we intend for the present results to serve as a benchmark against which we will search for any changes in the overall level of childhood diabetes prevalence and its spatial patterns.

### Addressing service needs in peri-urban and regional communities

A recent investigation surveying patients at 38 tertiary and regional Australian and New Zealand paediatric clinics caring for children with diabetes^30^ determined that over half of all study participants had glycaemic outcomes below recommended levels. However, management of childhood diabetes is challenging for many reasons and sub-optimal glycaemic outcomes at similar rates have also been observed in children from the urban-dominated ADDN network.^31^ Regional health care providers face unique challenges for service delivery, including a lack of specialist practitioners and general under-staffing problems,^30^ though effective models of care have been developed in this setting.^32^ Youths living outside of major urban centres in Australia have historically faced greater impacts of diabetes management on their life satisfaction at a similar level of glycaemic control.^33^ A recent study focussed on Australian adults in rural and remote settings reports encouraging results that that the level of services provided by multidisciplinary primary may be effective in compensating for the limited access to specialist care.^34^ In light of the concentration of diabetes childhood ‘hotspots’ in regional areas it is also reassuring that the intra-national variation of treatment delivery for diabetes remains an active topic of research in Australia.^35^

## Conclusions

We have successfully applied a statistical modelling procedure to derive novel small-area estimates of the geographical variation of youth diabetes prevalence across Australia from the 2021 Australian Census dataset. ‘Hotspots’ of elevated prevalence were identified in a number of regional areas, while lower prevalences were determined in the inner city zones of Australia’s largest cities.

## Ethics

Written informed consent is provided by carers of children newly diagnosed with T1D in Western Australia to have their data stored in the WACDD and allow their de-identified data to be used for research purposes. This study received ethics approval from the Child and Adolescent Health Service Human Research Ethics Committee (RGS0000002386), Western Australia.

## Funding

Associate Professor Ewan Cameron is funded under a People and Platforms award from the Stan Perron Charitable Foundation.

## Conflicts of interest

The authors declare the following financial interests/personal relationships that may be considered as potential competing interests: Ewan Cameron reports financial support was provided by Stan Perron Charitable Foundation. Peter Gething reports financial support was provided by Bill & Melinda Gates Foundation. Song Zhang reports financial support was provided by Bill & Melinda Gates Foundation. If there are other authors, they declare that they have no known competing financial interests or personal relationships that could have appeared to influence the work reported in this article.

## References

[1] Kraemer M.U., Hay S.I., Pigott D.M., Smith D.L., William Wint G.R., Golding N. (2016). Progress and challenges in infectious disease cartography. Trends Parasitol.

[2] Ostfeld R.S., Glass G.E., Keesing F. (2005). Spatial epidemiology: an emerging (or re-emerging) discipline. Trends Ecol Evol.

[3] Soltesz G., Patterson C.C., Dahlquist G., the EURODIAB Study Group (2007). Worldwide childhood type 1 diabetes incidence–what can we learn from epidemiology?. Pediatr Diabetes.

[4] Group, D.E.R.I. (1988). Geographic patterns of childhood insulin-dependent diabetes mellitus. Diabetes.

[5] Cardwell C., Carson D., Patterson C. (2006). Higher incidence of childhood-onset type 1 diabetes mellitus in remote areas: a UK regional small-area analysis. Diabetologia.

[6] Cardwell C., Carson D., Patterson C. (2007). Secular trends, disease maps and ecological analyses of the incidence of childhood onset Type 1 diabetes in Northern Ireland, 1989–2003. Diabet Med.

[7] Compés M.L., Feja C., De Guzman E.N., Aguilar I., Conde S., Alonso J.P. (2013). Bayesian analysis of the geographical variation of type 1 diabetes mellitus in under 15 yr olds in northeast Spain, 1991–2009. Pediatr Diabetes.

[8] Ball S.J., Haynes A., Jacoby P., Pereira G., Miller L.J., Bower C. (2014). Spatial and temporal variation in type 1 diabetes incidence in Western Australia from 1991 to 2010: increased risk at higher latitudes and over time. Health Place.

[9] Grundmann N., Mielck A., Seigel M., Maier W. (2014). Area deprivation and the prevalence of type 2 diabetes and obesity: analysis at the municipality level in Germany. BMC Public Health.

[10] McAlexander T.P., De Silva S.S.A., Meeker M.A., Long D.L., McClure L.A. (2022). Evaluation of associations between estimates of particulate matter exposure and new onset type 2 diabetes in the REGARDS cohort. J Expo Sci Environ Epidemiol.

[11] Joshi K.K., Haynes A., Smith G., Jones T.W., Davis E.A. (2018). Comparable glycemic outcomes for pediatric type 1 diabetes patients in metropolitan and non-metropolitan regions of Western Australia: a population-based study. Pediatr Diabetes.

[12] Haynes A., Curran J.A., Davis E.A. (2021). Two decades of increasing incidence of childhood-onset type 2 diabetes in Western Australia (2000–2019). Med J Aust.

[13] Australian Institute of Health and Welfare (2009). Diabetes prevalence in Australia: an assessment of national data sources (CVD46). https://www.aihw.gov.au/reports/diabetes/diabetes-prevalence-australia-assessment/summary.

[14] Lee A.E., Chiu C., Thian A., Suann B., Gorman S. (2022). Increased levels of solar radiation are associated with reduced type-2 diabetes prevalence: a cross-sectional study of Australian postcodes. Front Environ Sci.

[15] Statistics, A.B.o (1 Feb 2024). Long-term Health Conditions. https://www.abs.gov.au/articles/long-term-health-conditions#methodology.

[16] Statistics, A.B.o. (1 Feb 2024). Confidentiality and relative standard error. https://www.abs.gov.au/statistics/microdata-tablebuilder/tablebuilder/confidentiality-and-relative-standard-error.

[17] Cameron E. (2023).

[18] Scheme, N.D.S., Map of national diabetes services scheme (NDSS) registrants.

[19] SERIES, D. (2009).

[20] Diggle P.J., Ribeiro P.J., Christensen O.F. (2003). An introduction to model-based geostatistics. Spatial statistics and computational methods.

[21] Lawrence J.M., Divers J., Isom S., Saydah S., Imperatore G., Pihoker C. (2021). Trends in prevalence of type 1 and type 2 diabetes in children and adolescents in the US, 2001-2017. JAMA.

[22] Titmuss A., Korula S., Wicklow B., Nadeau K.J. (2022). Youth-onset type 2 diabetes among First Nations young people in northern Australia: a retrospective, cross-sectional study. Lancet Diabetes Endocrinol.

[23] Peña A.S., Curran J.A., Fuery M., George C., Jefferies C.A., Lobley K. (2020). Screening, assessment and management of type 2 diabetes mellitus in children and adolescents: Australasian Paediatric Endocrine Group guidelines. Med J Aust.

[24] Lopez-Doriga Ruiz P., Stene L.C. (2023). Is socio-economic status associated with risk of childhood type 1 diabetes? Literature review. Diabet Med.

[25] Payne E., Brown L.J., Crowley E., Rollo M., Schumacher T.L. (2020). Exploring core food accessibility in Tamworth, NSW, Australia. Inf Health Soc Care.

[26] D’Angeli M.A., Merzon E., Valbuena L.F., Tirschwell D., Paris C.A., Mueller B.A. (2010). Environmental factors associated with childhood-onset type 1 diabetes mellitus: an exploration of the hygiene and overload hypotheses. Arch Pediatr Adolesc Med.

[27] Wakefield J. (2008). Ecologic studies revisited. Annu Rev Publ Health.

[28] Runge J., Nowack P., Kretschmer M., Flaxman S., Sejdinovic D. (2019). Detecting and quantifying causal associations in large nonlinear time series datasets. Sci Adv.

[29] Clapin H., Phelan H., Bruns L., Sinnott R., Colman P., Craig M. (2016). Australasian diabetes data network: building a collaborative resource. J Diabetes Sci Technol.

[30] de Bock M., Jones T.W., Fairchild J., Mouat F., Jefferies C. (2019). Children and adolescents with type 1 diabetes in Australasia: an online survey of model of care, workforce and outcomes. J Paediatr Child Health.

[31] James S., Perry L., Lowe J., Harris M., Craig M.E., the ADDN study group (2022). Suboptimal glycemic control in adolescents and young adults with type 1 diabetes from 2011 to 2020 across Australia and New Zealand: data from the Australasian Diabetes Data Network registry. Pediatr Diabetes.

[32] Goss P.W., Paterson M.A., Renalson J. (2010). A ‘radical’new rural model for pediatric diabetes care. Pediatr Diabetes.

[33] Cameron F., Clarke C., Hesketh K., White E.L., Boyce D.F., Dalton V.L. (2002). Regional and urban Victorian diabetic youth: clinical and quality-of-life outcomes. J Paediatr Child Health.

[34] Skinner T., Allen P., Peach E., Browne J.L., Pouwer F., Speight J. (2013). Does the shortage of diabetes specialists in regional and rural Australia matter? Results from Diabetes MILES—Australia. Diabetes Res Clin Pract.

[35] Carrigan A., Lake R., Zoungas S., Huynh T., Couper J., Davis E. (2022). Mapping care provision for type 1 diabetes throughout Australia: a protocol for a mixed-method study. BMJ Open.

