# Supplementary material for: Small-area geographical variation in the prevalence of diabetes amongst Australian youth aged <20 years in 2021
Source: Aust N Z J Public Health. 2025 Jun;49(3):100234. doi: 10.1016/j.anzjph.2025.100234 (PMC12170347; doi:10.1016/j.anzjph.2025.100234)
Supplement: Multimedia component 1 [file mmc1.docx]

# Supplementary Information

*Statistical model*. In each SA1 unit ($i=1,\ldots,N_{SA1}$) and age group ($a=1, 2, 3$) the count of positive responses, $k_{i}^{a}$, to the “Has diabetes (excluding gestational diabetes)” prompt in the long-term health conditions question is modelled as a Binomial random variable given the total response count, $n_{i}^{a}$, and an (unknown) underlying prevalence, $p_{i}^{a}$; that is, $k_{i}^{a}\sim\mathrm{Binomial}(\mathrm{mean}= p_{i}^{a}, \mathrm{total}=n_{i}^{a})$. The underlying prevalence vector, $p^{a}$, i.e., the vector of rates enumerated across all SA1 units: $p^{a}=p_{i=1,\ldots,N_{\mathrm{SA}1}}^{a},$ is the target of our inference procedure.

We create a hierarchical Bayesian model for $p^{a}$by supposing for our youngest and oldest age groups (i.e., $a=1, 3$) that the logit transform of $p^{a}$ in each case is formed by the sum of an intercept, a socio-economic effect, a remoteness effect and a spatially-correlated random field. For $a=1, 3$ (meaning children aged 0–9 and 14-19 years old, respectively) we specify that

$$\mathrm{logit} p^{a}=\alpha_{a}+Y_{SES, a}+Y_{RA, a}+Z_{a}$$

with

$${Y_{SES, a}\sim\mathrm{AR}_{\xi_{a}}(0.9), Y_{RA,a}\sim\mathrm{AR}_{\chi_{a}}(0.9), Z}_{a}\sim\mathrm{GP}_{\theta_{a},\phi_{a}}$$

and $\alpha_{a}$ assigned an improper uniform prior. Here $Y_{SES, a}$ and $Y_{RA, a}$ are modelled as realisations of auto-regressive processes with characteristic parameters of 0.9 (fixed to this value close to one to approximate moving average processes without the numerical instabilities) and scale parameters of $\xi_{a}$ and $\chi_{a}$, respectively. The latter are assigned hyper-priors of $\log\xi_{a}\sim\mathrm{Normal}\left( 1,1 \right)$ and $\log\chi_{a}\sim\mathrm{Normal}\left( -1,1 \right)$. is a spatial random field (i.e., a two dimensional Gaussian process over Cartesian longitude-latitude space). A Matérn covariance function (of $\nu=0.5$ smoothness) is adopted for the spatial random field with range, $\theta_{a}$, and scale, $\phi_{a}$, parameters assigned hyper-priors of $\log\theta_{a}\sim\mathrm{Normal}\left( 1,1 \right)$ and $\log\phi_{a}\sim\mathrm{Normal}\left( -1,1 \right)$. For $a=2$ (meaning children aged 10–14 years old) we suppose that $\mathrm{logit} p^{2}$ is the mixture of $\mathrm{logit} p^{1}$and $\mathrm{logit} p^{3}$ in proportions of $\gamma$ and $1-\gamma$, respectively, plus an intercept offset of $\alpha_{2}$.

The computational implementation of this model is made numerically tractable through a series of approximations. First, the random fields are represented using the mesh-based stochastic partial differential equation structure of the INLA package for R. Second, the marginal likelihood at a given choice of the hyper-parameters is estimated via the Laplace approximation in the TMB package for R. And third, the posterior itself is summarised by expansion about the marginal posterior mode of the hyper-parameters. The R and TMB code used for this analysis is made publicly available online at https://github.com/drewancameron/diabetesmodel .

The posterior median and 95% credible interval for the $\gamma$ parameter defining the mixing proportion of the youngest and oldest age group trends for mapping prevalence in the middle age group was found to be 0.98 [0.93–0.99]. Posterior summaries for the random effects are given in Supplementary Tables 1 and 2 below.

|  | IRSAD 2 | IRSAD 3 | IRSAD 4 | IRSAD 5 | IRSAD 6 | IRSAD 7 | IRSAD 8 | IRSAD 9 | IRSAD 10 |
| --- | --- | --- | --- | --- | --- | --- | --- | --- | --- |
| 0-9 year olds | -0.11 [-0.21,-0.01] | -0.14 [-0.24,-0.04] | -0.21  [-0.32,-0.10] | -0.24  [-0.35,-0.14] | -0.21  [-0.32,-0.10] | -0.27  [-0.38,-0.16] | -0.31  [-0.43,-0.20] | -0.39  [-0.52,-0.26] | -0.37  [-0.51,-0.23] |
| 15-19 year olds | -0.04 [-0.12,0.05] | -0.10 [-0.20,-0.01] | -0.15  [-0.24,-0.05] | -0.18  [-0.28,-0.08] | -0.23  [-0.33,-0.13] | -0.25  [-0.35,-0.15] | -0.30  [-0.41,-0.19] | -0.32  [-0.44,-0.21] | -0.33  [-0.45,-0.21] |

**Supplementary Table 1** Posterior median and 95% credible interval of the socio-economic effect for the youngest and oldest age groups in our model. Here the lowest IRSAD decile (not shown) is our reference category (fixed to zero).

|  | Remoteness Area 2 | Remoteness Area 3 | Remoteness Area 4 | Remoteness Area 5 |
| --- | --- | --- | --- | --- |
| 0-9 year olds | 0.06 [-0.04,0.14] | -0.01 [-0.12,0.10] | -0.05  [-0.21,0.12] | 0.02  [-0.23,0.09] |
| 15-19 year olds | 0.08 [-0.01,0.16] | 0.04 [-0.06,0.5] | 0.15  [-0.02,0.33] | 0.31  [0.09,0.53] |

**Supplementary Table 2** Posterior median and 95% credible interval of the remoteness effect for the youngest and oldest age groups in our model. Here the “Major city” Remoteness Area code (not shown) is our reference category (fixed to zero).
